# [Retracted] MicroRNA‑17‑5p contributes to osteoarthritis progression by binding p62/SQSTM1

**DOI:** 10.3892/etm.2024.12653

**Published:** 2024-07-15

**Authors:** Huihui Li, Daoyi Miao, Qi Zhu, Jianghua Huang, Guangxian Lu, Weiguo Xu

Exp Ther Med 15:1789–1794, 2018; DOI: 10.3892/etm.2017.5622

Following the publication of this paper, it was drawn to the Editor’s attention by a concerned reader that the western blotting data shown in Fig. 3C and D on p. 1792, in addition to the style of presentation of the diagram showing the binding site between miR-17-5p and p62 in Fig. 3A, were strikingly similar to the contents of Fig. 2 in a different article written by different authors at different research institutes, which had already been submitted for publication one month in advance of the above paper to the journal *Die Pharmazie*.

Owing to the fact that the above-mentioned data had already been submitted for publication prior to its submission to *Experimental and Therapeutic Medicine,* the Editor has decided that this paper should be retracted from the Journal. The authors were asked for an explanation to account for these concerns, but the Editorial Office did not receive a reply. The Editor apologizes to the readership for any inconvenience caused.

